# Quantitative Analysis of Different Environmental Factor Impacts on Land Cover in Nisos Elafonisos, Crete, Greece

**DOI:** 10.3390/ijerph17186437

**Published:** 2020-09-04

**Authors:** Mohamed Elhag, Silvena Boteva

**Affiliations:** 1 Department of Hydrology and Water Resources Management, Faculty of Meteorology, Environment and Arid Land Agriculture, King Abdulaziz University, Jeddah 21589, Saudi Arabia; 2 Department of Applied Geosciences, Faculty of Science, German University of Technology in Oman, Muscat 1816, Oman; 3 Department of Ecology and Environmental Protection, Faculty of Biology, Sofia University, 1164 Sofia, Bulgaria; silvenab@abv.bg

**Keywords:** cross-tabulation, environmental clusters, Mediterranean ecosystems, S2REP, LULC

## Abstract

Land Cover monitoring is an essential task for a better understanding of the ecosystem’s dynamicity and complexity. The availability of Remote Sensing data improved the Land Use Land Cover mapping as it is routine work in ecosystem management. The complexity of the Mediterranean ecosystems involves a complexity of the surrounding environmental factors. An attempt to quantitatively investigate the interdependencies between land covers and affected environmental factors was conducted in Nisos Elafonisos to represent diverse and fragile coastal Mediterranean ecosystems. Sentinel-2 (MSI) sensor and ASTER Digital Elevation Model (DEM) data were used to classify the LULC as well as to draw different vegetation conditions over the designated study area. DEM derivatives were conducted and incorporated. The developed methodology is intended to assess the land use land cover for different practices under the present environmental condition of Nisos Elafonisos. Supervised classification resulted in six different land cover clusters and was tested against three different environmental clusters. The findings of the current research pointed out that the environmental variables are independent and there is a vertical distribution of the vegetation according to altitude.

## 1. Introduction

Studying and understanding the vegetation and land use pattern is of great importance for the understanding of many ecological processes and the functioning of complicated systems such as landscape. Verburg et al. [1] found that the land use pattern of a region has a strong influence on a variety of ecological phenomena such as net primary production. The Land Use Land Cover (LULC) pattern creates new processes, influencing, for example, horizontal movement and distribution of animal populations [2,3], water runoff and erosion (e.g., [4], the spread of disturbance [5,6], and fluxes of materials and energy [7] or boundary phenomena in general [8]).

The LULC composition and changes are important factors that affect the ecosystem’s condition and its functionality. These factors are frequently used to generate landscape-based metrics and to assess landscape conditions and monitor status and trends over a specified time interval [9]. The use of optical remote sensing imagery has been widely applied to provide a cost-effective means to develop LULC coverage’s over large geographic regions [10,11].

One important method of understanding ecological dynamics, such as natural and human disturbances, ecological succession, and recovery from previous disturbances, is the analysis of changing landscape patterns [12,13]. Satellite imagery and aerial photography that have been classified by vegetation type provide an excellent source of data for performing structural studies of a landscape [14,15].

Simple measurements of patterns, such as the number, size, and shape of patches, can indicate more about the functionality of a land cover type than the total area of cover alone [16,17]. When fragmentation statistics are compared across time, they are useful in describing the type of landscape change and indicating the resulting impact on the surrounding habitat [18,19].

Vegetation indices are optical remote sensing offshoots to assess and to monitor the vegetation vigorously on a regional scale. There are over 20 vegetation indexes developed to estimate the vegetation conditions from different aspects and for different purposes [20,21]. Optical remote sensing data obtained from Sentinel-2 Multispectral Instrument (MSI) has the lead in vegetation indices estimation due to the red edge channel [21,22]. Sentinel-2 Red Edge Position Index (S2REP) is one of the most advanced indices in assessing the vegetation conditions under the linear interpolation of MSI red-edge bands 5 and 6 [23,24]. According to Birgin and Martínez [25], this method has the key benefit over the Lagrangian method of reflectance measurements at the inflection point due to the limited number of spectral bands [26,27,28].

Quantitative analysis is used to delineate the relationships between environmental factors and Land Use Land Cover units. They are clearly displayed in matrices which contain the description of objects, at the same time or at different times by variables that might have been measured in different scales and clusters. This level of classification detail presents opportunities for analyzing landscape change patterns at a structural scale [29,30]. Cluster analysis and discrimination analysis techniques are used to explore the similarities between objects and to define groups of objects by considering simultaneously all the measured variables [31,32]. There are two major thrusts in mathematical modeling within GIS environments: Optimization and simulation [33,34]. Each represents a fundamentally different approach to problem-solving. Broadly speaking, the output of optimization models is a prescription of strategy. Simulation, on the other hand, is a descriptive approach.

The use of geoformation tools to envisage the interpretation of spatial relationships between environmental parameters as independent variables and Land Use Land Cover units as dependent variables were developed under the Geographical Information System (GIS) environment. Geostatistical, density, and buffer analysts were the most exercised tools in environmental management issues when natural resources were specifically considered [35,36].

The landscape of Nisos Elafonisos is of a high aesthetic and great natural ecosystem. The various ecotopes coexist and complete each other creating a diverse entity. Human intervention is obvious all over the surrounding area. The tourist development started three decades ago. Some projects are referring to the parts of the area or the whole of it but none of them studies the area from a landscape point of view, as an entity of the four dominant landscapes [37]. Therefore, the main objective of the current study is to understand the combined influences of the environmental factors, and the human intervention has determined the vegetation distribution using the enclosed environmental factors and the Land Use Land Cover variabilities. A study like this would be very useful in order to create a general image of the particular area as a fully integrated landscape and to reveal the changes and the trends of the landscape through time. The study can be used for future research in conservation and planning of the land uses of the area.

## 2. Materials and Methods

### 2.1. Study Area Description

**Figure 1:**
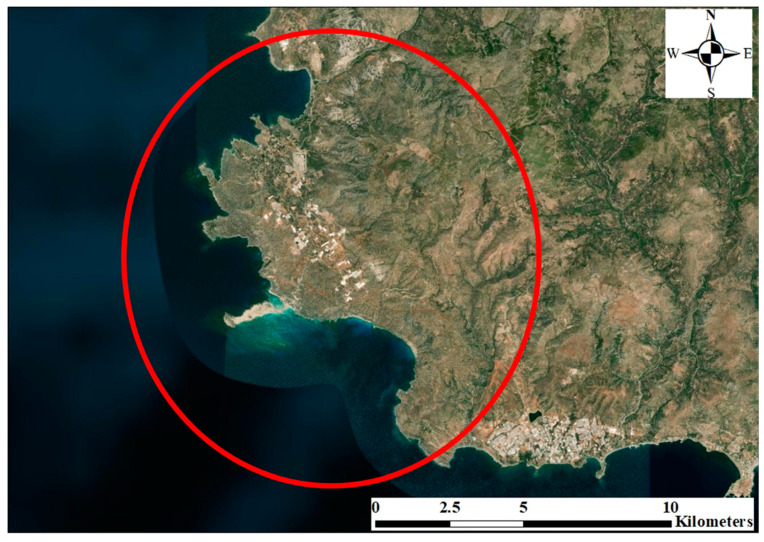
The location of the study area in Crete Island, Greece.

The study area, Nisos Elafonisos, is located in Southwest of Crete and covers an area of about 4317.21 ha; as is enclosed by the red box illustrated in Figure 1. The area is affected by Mediterranean weather conditions. There are two main seasons: Dry hot summer and rainy cold winter. The rain season starts in October and ends in April of the next year. The dry season starts in June and ends in September of the same year. The average temperature recorded from 1972 to 2012 is about 18 °C. The mean annual rainfall is about 750 mm. The topography of the area is considered to be moderate lowlands. Sclerophyllous vegetation is the dominant land cover with a small area used for olive groves. The study area experienced heavy tourism activities throughout the last decade.

### 2.2. Data Processing

The process of evaluating the land clusters is adopted from the FAO framework developed by Verheye, Koohafkan [38]. The method to be proposed is intended to design for assessing land for different practices under the present condition in Nisos Elafonisos. In order to develop a set of themes for evaluation and ultimately to produce a suitability map, the condition requirement in terms of land qualities and land topography was reviewed [39].

Cluster analysis aims to place objects into groups or clusters suggested by the dataset, not defined a priori. Consequently, the objects in each cluster tend to be similar, and objects in different clusters tend to be dissimilar. For the purpose of this study, a hierarchical cluster procedure which attempts to identify relatively homogeneous groups of cases based on selected variables, more specifically, Ward’s method, based on the sum of the squared differences between the values for the items, is applied to classify the designated study area. The results of the classification are then displayed in the form of maps that show the spatial distribution of the Operational Geographic Unit (OGU) classes. According to Murtagh and Legendre [40], Ward’s Error sum of squares method was conducted as follows:

$$ESS=\underset{iЄq}{\sum }d^{2}\left(i,q\right)$$

where

*d* is the absolute distance between to the two events *i*,*q*.

The temporal data set was downloaded from the European Space Agency (ESA) data hub. The first dataset was acquired in March 2014 and the second dataset was acquired in March 2019. The remote sensing data were radiometrically and atmospherically corrected according to [41,42]. Support Vector Machine (SVM) classifier was exercised on the temporal data sets to obtain to Land Clover Land Use classes according to Chavez [43] in a very simplified form as follows:

$$K\left(x_{i},x_{j}\right)=tanh\left(gx_{i}^{T}x_{j}+r\;\right)$$

where:

*g* is the kernel function gamma term for all kernel types except linear
*r* is the kernel function bias term for the polynomial and sigmoid kernels.
*T* is the kernel Trick (the bridge from linearity to non-linearity to any algorithm).

To assess the vegetation cover of the study area temporally, Sentinel-2 Red-Edge Position Index algorithm was applied according to Frampton et al., as follows:

$$S2REP=705+35*\frac{\left(\frac{B4+B7}{2}-B5\right)}{B6-B5}$$

where B is the band information shown in Central Sentinel-2 wavelength/Bandwidth

*B*7 = 783 nm (15 nm),
*B*6 = 740 nm (15 nm),
*B*5 = 705 nm (15 nm),
*B*4 = 665 nm (30 nm).

Classification accuracy assessment was carried out according to Congalton and Mead [44] as follows:

$$K_{hat}=\frac{N\cdot \sum_{i=1}^{r}x_{ii}-\sum_{i=1}^{r}\left(x_{ij}\cdot x_{ji}\right)}{N^{2}-\sum_{i=1}^{r}\left(x_{ij}\cdot x_{ji}\right)}$$

where

*r*, is the number of rows in the error matrix
*x_ii_*, is the number of observations in row i and column i (the diagonal cells)
*x_i+_*, is the total observations of row i
*x_+I_*, is the total observations of column i
*N*, is the total of observations in the matrix

The land qualities to be used in this evaluation thus include several land characteristics layers, for this study area soil-geology, slope, precipitation, temperature, and S2REP change detection layer from the temporal satellite images in addition to the DEM layer (Table 1). Each land characteristic is considered a thematic layer under the GIS environment. In order to create an environmental dataset, the following steps have been performed.

In order to create the dependence matrix of Land Use Land Cover dataset, almost the same procedure was repeated by cross tabulating land cover with land units and taking, as a result, a matrix with land cover types in one side and the land units in the other side and then they were standardized in percentage (%) as shown in Table 3.

By joining all the reclassified maps with the land cover types, the matrix which includes dependent and independent variables was ready, and every land unit was given this information. The geographic units were classified twice; depending on the land cover types (6 groups) and depending on environmental parameters (3 groups) as listed in Table 4.

Finally, the two matrices were tabulated to draw some conclusions. To make the analysis clear, one tabulation step takes place between the groups, and another one between the variables themselves was done. The number of clusters of the land cover is expanded because they are too heterogeneous, so six clusters are created for the land cover which is described by 15 land cover/land use types. Now, these six clusters must be characterized according to the environmental variables.

## 3. Results

GIS environment was used to reclassify the grouped landscape units to see the spatial relationship between these clusters for both data sets and they were cross-tabulated to see the overlap (Common area). The variables of six clusters are grouped depending on environmental variables. Two maps were created which were classified into three classes for the environmental clusters and in six classes for land cover variables.

**Figure 2:**
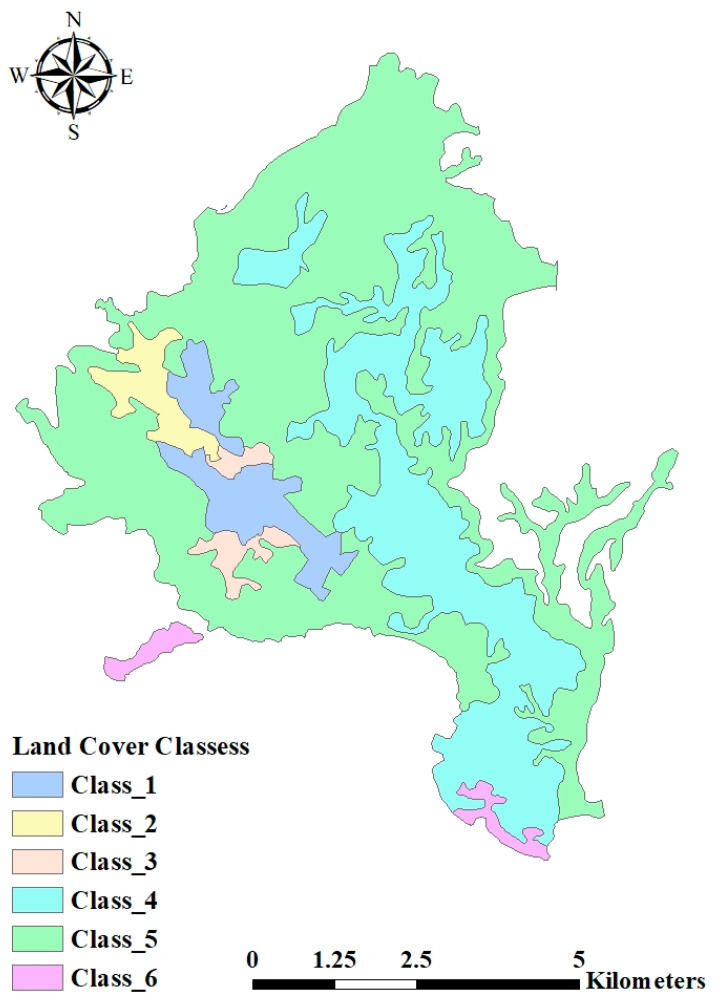
Supervised Land Cover Land Use classes of the designated study area.

**Figure 3:**
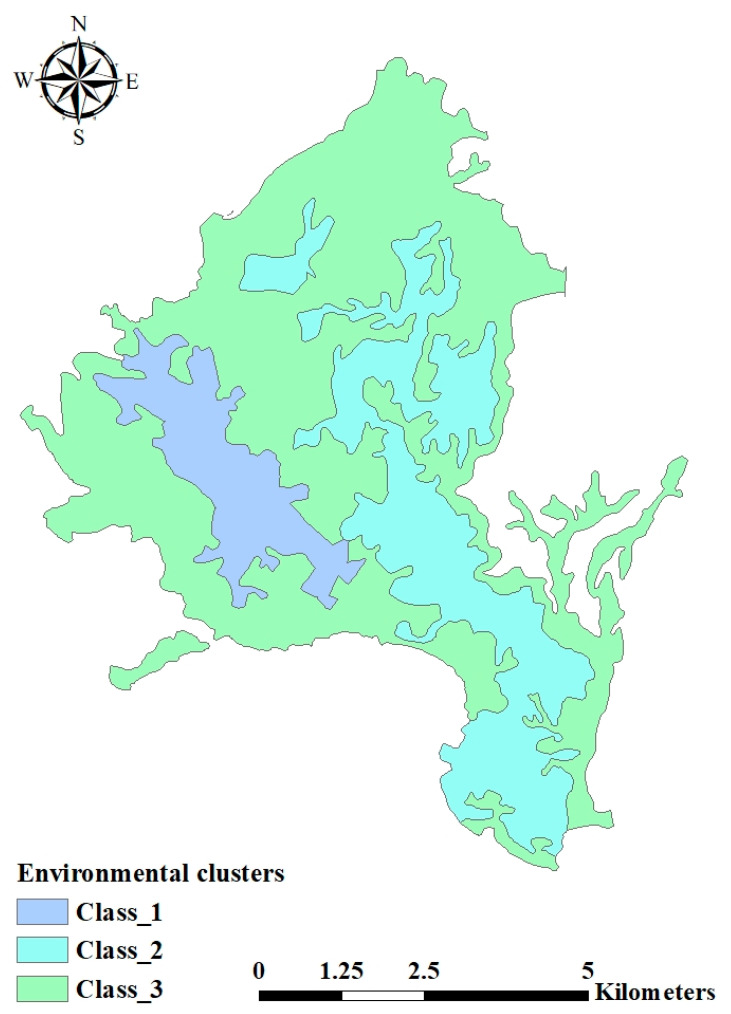
Environmental clusters of the designated study area.

Figure 2 and Figure 3 showed the map of the study area classified into groups according to the dependent and independent variables. To get the description of each land cover cluster according to environmental factors, the percentages of the overlap between land cover and environmental clusters were calculated as shown in Table 5.

The fuzzy set represents the membership of an object to a specific cluster, which leads to differentiate the clusters according to the objects falling in each of them. This membership defined by the distance between an object from the centers of the clusters. Using the similarity matrices of dependent and independent variables, the variability of the dependent variables based on the independent variables can be described. The geographic units in the matrix of the independent variables were rearranged according to the clusters of the land cover matrix. The structure of the matrix of similarity based on environmental variables according to Land Use Land Cover is illustrated in Table 6.

**Figure 4:**
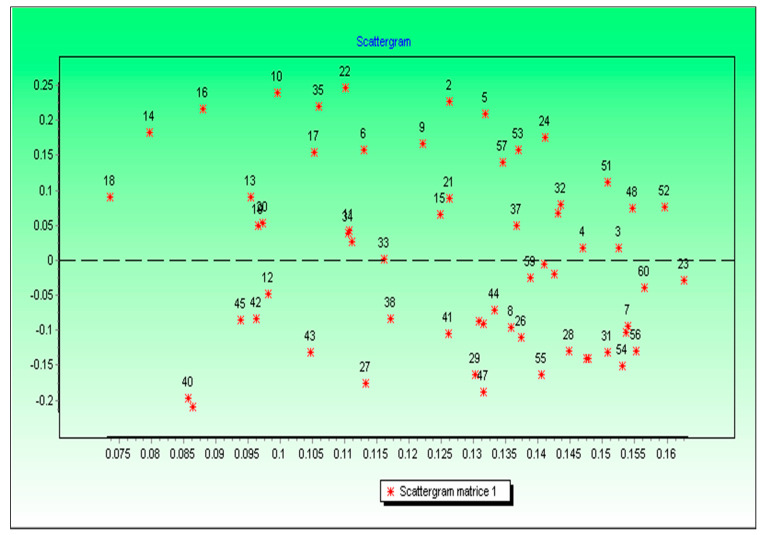
The scattergram matrix environmental variables.

Matching to the maps in Figure 2 and Figure 3 and the values in Table 6 the following clusters could be concluded according to the scattergram matrix (Figure 4):

Distances are computed among the considered objects, based on the standardized values of all the parameters, the standardization method removes the effects of the scale and the measure of the data. After performing the join between the created database, containing the cluster’s membership scores, and the communes map, the range of solutions, are visualized under GIS environment, and regarding the previous knowledge from data correlation and other analysis, the solution of 6 clusters is chosen:

**First cluster:**

The first land cover cluster overlaps with the first cluster of environmental variables by 91.70%. Thus, a strong relationship exists between this land cove type and environmental variables. It can be noticed that this land cover group consists mainly of shrubs and **grassland vegetation** distributed in the regions with the following environmental conditions:

- Mainly in SE-SW and NE-NW aspects,
- High elevation; greater than 1000 m,
- Steep slope about 33°, and
- Increase in S2REP values.

**Second cluster:**

Of the second land covers cluster, 98.7% overlap with the first cluster of environmental variables while the remaining 1.3% is located in the second one. According to the tables in the appendix, this group mainly consists of **broad leaves and mixed forest** located in the following environmental conditions:

- SE_SW aspects,
- High elevation (greater than 1000 m) with a considerable percent about 91%,
- Wide range slope starting with flat areas to steep ones, and
- Increase in S2REP values.

**Third cluster:**

The third cluster consists of **industrial, heterogeneous agriculture, and shrubs areas** with an overlap of about 23.87% and 76.13% with the first and the second environmental clusters, respectively. This area is valuable with an environmental perspective because of the following properties:

- Low elevation,
- Low slope from 0 to 10°, and
- A decrease in S2REP values.

**Fourth cluster:**

The fourth land cover cluster is not strongly correlated with any environmental cluster. However, it is distributed in the first, second, and third environmental clusters with 12.65%, 29.82%, and 57.53%, respectively. It occupied mainly by **broad-leaved forest** and partially by **shrublands** with the following environmental parameters:

- SE-SW aspects,
- Medium elevation,
- Moderate climatic conditions, and
- Moderate slope.

**Fifth cluster:**

This cluster is mainly occupied by **arable land** and it strongly overlaps with the second environmental cluster with 63.24%. The following environmental conditions can be noticed

- A low slope or nearly flat areas,
- Low elevation, and
- A decrease in S2REP values.

**Sixth cluster:**

In this cluster, the wetland area with very little vegetation can be found, and it strongly correlated to the second environmental clusters. The elevation in this area is considered low.

## 4. Discussion

Mapping the environmental factors in accordance with the Land Use Land Cover dynamicity is a mandate to sustain the fragile ecosystem of Nisos Elafonisos. Such balance will be generally based on the vast unpredictability of the primary elements of the designated ecological system [45,46]. The environmental restoration of natural areas will be possible when their natural and biological capacities are regularly monitored [47]. Consequently, several zoning/clustering approaches have been formulated and implemented along the past decades as the methods developed synchronously with the advancement of science and technology development [48,49].

Understanding Land Use Land Cover change requires an integrated investigation of human and ecological driving processes and responses to change. Patterns of land-use/cover change result from the interactions between human and natural factors and imprint their legacies on the landscape in patterns that are distinctive and detectable. According to Blaschke [50], human land use has influenced most landscapes, resulting in a landscape mosaic of natural and human-managed patches that vary in size, shape, and arrangement. Remote sensing and Geographic Information Systems (GIS) are valuable tools for detecting, monitoring, and modeling landscape changes. On the other hand, indices of landscape structure and function provide important information about the development and dynamics of landscape patterns and how they relate to ecological phenomena. Some common questions are focused upon the relationship between the changes that occur in the landscape and the spatial configuration of landscape attributes. Numerical and spatial data processing is needed to quantify and analyze these historical spatial patterns of Land Use Land Cover.

Clustering proceeds by pairing nearest objects, which are the most similar, to build up a group of objects until all have been grouped into clusters Classical clustering approaches generate partitions such that each object is assigned to exactly one cluster. Often, however, objects cannot adequately be assigned to strictly one cluster (because they are located “between” clusters). In these cases, fuzzy clustering methods provide a more adequate tool for representing data structures [51].

In the numerical and spatial analyses of land mosaic, two types of information are processed: The patch attributes such as size, shape, and spatial arrangements and the landscape attributes [52]; and the analyses for understanding the complexity of the land mosaic. Some of the basic measures of patch characteristics are patch size, patch perimeter, and patch shape. According to Perry et al. [53] and Elhag [54], the shape, format, and size of patterns most likely reflect the ecological status of a place and therefore represent an easy and certain way to distinguish areas on the earth.

Chamapira, and Taghavi [55] suggested that the Operational Geographic Unit dimension captured some aspect of the surface roughness that was unique. A spatial roughness pattern could be derived directly from the spectral imagery and it should be useful for further spatial analysis within GIS environments. Several different methods including the dividers method, cell (box) counting method, and variogram method were compared for determining the Operational Geographic Unit dimension of topographic surfaces [56,57]. They concluded that it provided a fairly complete descriptor for some landscapes although it couldn’t be considered a universal model It would appear that the variability in Operational Geographic Unit dimension is more a function of the methods used to obtain the unit dimension than it is a reflection of any theoretical inadequacy of self-similar units model [58,59].

## 5. Conclusions and Recommendations

Environmental variables were used as the independent variables because they determine mainly the vegetation distribution and land use of the area, which can further be changed by human intervention. The environmental variables are independent because they are stable for long periods and are not sensitive to human interference. These variables were grouped into three classes according to their similarity. The land cover was the dependent variable, the one that we were trying to analyze through some other independent variables known as the Environmental variables. The land cover was better discriminated in six clusters. By using the summarized information for each class, we can draw the following conclusion: There is a vertical distribution of the vegetation according to altitude. From these analyses, we can conclude that the areas that are covered by forests are small, compared to the whole area. The forests are mainly found in the first three clusters located in high altitudes, far from human interference. It can be noticed that stands of coniferous forest cover only a very small percentage of the area. Moving from high elevation to low elevation there is a clear change in land cover. In high elevation most of the area is occupied by shrubs and forest, with some open spaces. The human interference is low in high elevation due to the environmental limitation, only in the case of the open spaces: This probably comes as a result of clear-cutting of forests. The physical limitations of the environment confine somehow the human interference as a result of high altitudes, a long distance from the urban areas, high slopes and a decrease in S2REP values which make these areas unsuitable for human activities such as cultivation or industry. But moving from upper to lower elevation, it starts to appear the human interference, the arable land appears in the second cluster but belonging to this cluster in a very small percentage and anyway in low elevation. The vegetation cover is reduced with altitude; in lower elevations, the mixed forest disappears, while it starts to be more frequent on the arable land. This is explained by favorable environmental conditions: Low slopes, and low elevation, which makes easy the cultivation of such areas.

## Figures and Tables

**Table 1:** Reclassification of the natural parameters into different classes.

Variable		Classes
**Vegetation**	1	Natural grasslands
	2	Complex cultivation patterns
	3	Sclerophyllous vegetation
**Habitats**	4	Agricultural land
	5	Formations composed mostly or predominantly of annuals, in particular, Chenopodiaceae
	6	Juniper formations of Mediterranean coastal dune slacks and slopes, J. communis formations
	7	Large indentations of the coast where, in contrast to estuaries, influence by freshwater is limited
	8	Low, thorny formations of hemispherical shrubs of the coastal thermo-Mediterranean zone
	9	The Mediterranean and thermo-Atlantic woods of thermophilous pines
	10	Mediterranean humid grasslands of tall grasses and brushes
	11	Meso- and thermo-Mediterranean xerophile, short-grass annual grasslands rich in therophytes
	12	Moving sand dunes, formed in the line of undulation or coastal sand dunes systems
	13	Sclerophyllous scrubs established on dunes of the Mediterranean regions
	14	Tamarisk, oleander and chaste tree galleries and similar low ligneous formations of permanent
	15	Thermo-Mediterranean woodland dominated by arborescent Olea europaea ssp. sylvestris
	16	Vegetated cliffs and rocky shores of the Mediterranean
	17	Vegetation found in calcareous declivities
	18	Very shallow temporary ponds (a few cms deep) which exist only in winter or late spring
	19	Woods, often riparian, formed by the palm Phoenix theophrasti, restricted to sandy coastal valleys
	20	Woody coppice mainly consisting of Juniperus phoenicea.
**Geology**	21	Mixed formation
	22	Stavros-Seli schists
	23	Dolomites, dolomitic limestones, limestones
	24	Flysch
	25	Limestones
	26	Recrystallized limestones and dolomites
	27	Talus cones and scree
	28	Transition beds
	29	Undivided neogene formations
**Soil**	30	Arenosoils
	31	Cambisoils
	32	Gleysoils
	33	Lithosoils
	34	Luvisoils
	35	Rankers
	36	Regosoils
	37	Solonetz
	38	Terra rossa
	39	Vertisoils
**Elevation**	40	20
	41	60
	42	100
	43	140
	44	180
**Slope °**	45	0
	46	10
	47	20
	48	40
	49	60
**Aspect**	50	North
	51	Northeast
	52	East, northwest
	53	West, southeast
	54	Southwest
	55	South
**Temperature**	56	19
	57	20
	58	21
	59	22

Consequently, cross-tabulation of DEM derivations with the S2REP temporal values was conducted resulting in a matrix with environmental variables for each unit. The variables are standardized into percentages (%). Environment variables are in the columns versus landscape units in the rows as shown in Table 2.

**Table 2:** Cross-tabulation of Digital Elevation Model (DEM) products and Sentinel-2 Red Edge Position Index (S2REP).

Elevation (m)				Slope				Aspect					S2REP	
>100	100–350	350–700	<700	5°	15°	35°	60°	N	NE–NW	E–W	SE–SW	S	−ve	+ve
0.00	0.00	0.00	1.00	0.04	0.18	0.68	0.10	0.14	0.25	0.29	0.13	0.20	0.00	1.00
0.00	0.00	0.00	1.00	0.07	0.18	0.68	0.07	0.17	0.09	0.25	0.21	0.29	0.00	1.00
0.00	0.00	0.17	0.83	0.08	0.21	0.62	0.09	0.21	0.12	0.21	0.22	0.24	0.14	0.86
0.00	0.01	0.16	0.84	0.08	0.19	0.58	0.15	0.23	0.08	0.15	0.20	0.34	0.07	0.93
0.00	0.00	0.09	0.91	0.11	0.14	0.56	0.19	0.16	0.15	0.21	0.19	0.29	0.15	0.85
0.00	0.00	0.17	0.83	0.04	0.09	0.59	0.28	0.15	0.11	0.20	0.24	0.30	0.08	0.92
0.00	0.00	0.19	0.81	0.06	0.21	0.68	0.05	0.30	0.06	0.13	0.15	0.36	0.00	1.00
0.00	0.00	0.18	0.82	0.08	0.26	0.63	0.04	0.25	0.06	0.15	0.20	0.34	0.02	0.98
0.00	0.00	0.23	0.77	0.04	0.19	0.72	0.05	0.24	0.02	0.10	0.22	0.42	0.01	0.99
0.00	0.03	0.24	0.73	0.04	0.12	0.61	0.23	0.12	0.15	0.29	0.23	0.22	0.09	0.91
0.00	0.03	0.27	0.70	0.09	0.16	0.56	0.19	0.08	0.15	0.30	0.25	0.22	0.17	0.83

E is East, W is West, SE is Southern East, SW is Southern West, S is South -ve is a negative S2REP value, +ve is a positive S2REP value.

**Table 3:** Cross-tabulation of Land Cover Land Use classes.

Land Use Land Cover												
Shrubland	Beach	Bare Land	Olive Grove	Urban Area	Grassland	Arable Land	Wetland	Industrial	Complex Vegetation	Mixed Forest	Pasture	Scrubland
0.06	0.40	0.11	0.29	0.11	0.02	0.00	0.00	0.00	0.00	0.15	0.18	0.00
0.04	0.15	0.29	0.24	0.05	0.13	0.03	0.01	0.00	0.00	0.23	0.29	0.01
0.20	0.18	0.10	0.30	0.06	0.07	0.01	0.00	0.00	0.00	0.20	0.26	0.01
0.07	0.12	0.19	0.36	0.02	0.05	0.01	0.00	0.00	0.00	0.26	0.34	0.01
0.10	0.04	0.04	0.01	0.01	0.20	0.12	0.01	0.01	0.00	0.16	0.20	0.01
0.02	0.09	0.42	0.07	0.03	0.11	0.10	0.00	0.06	0.00	0.13	0.14	0.01
0.05	0.00	0.00	0.00	0.00	0.00	0.00	0.19	0.25	0.17	0.14	0.23	0.01
0.08	0.00	0.00	0.00	0.00	0.00	0.00	0.10	0.21	0.22	0.16	0.26	0.01
0.06	0.12	0.00	0.00	0.00	0.00	0.00	0.16	0.24	0.22	0.56	0.64	0.01
0.37	0.01	0.02	0.01	0.00	0.01	0.05	0.13	0.21	0.21	0.22	0.29	0.02
0.07	0.00	0.01	0.01	0.00	0.00	0.00	0.07	0.17	0.21	0.15	0.18	0.00

**Table 4:** Dependent and independent variables enclosed within the study area.

Dependent Variables	Independent Variable
>100	Shrubland
100–350	Beach
350–700	Barren land
<700	Olive groves
5°	Urban
15°	Grassland
33°	Arable land
60°	Wetland
North	Industrial area
North-East, North-west	Complex vegetation
East, West	Mixed forest
South-East, South-west	Pastures
South	Scrub and/or herbaceous vegetation associations
	Decrease in S2REP
	Increase in S2REP

**Table 5:** The overlap percentages between the environmental cluster and Land Use Land Cover classes.

Classes	Environmental Cluster-1	Environmental Cluster-2	Environmental Cluster-3
**Land Cover-1**	91.7	6.5	1.8
**Land Cover-2**	98.7	1.3	0
**Land Cover-3**	23.87	76.13	0
**Land Cover-4**	12.65	29.82	57.53
**Land Cover-5**	63.24	36.76	0
**Land Cover-6**	0	100	0

**Table 6:** The Land Use Land Cover similarity matrix.

No. of Polygon	Arable Land	Bare Land	Beech	Grassland	Olive Groves	Shrub Land	Urban	Wet Land	
61	0.799906	0.58718	0.431012	0.768129	0.674134	0.632161	0.777378	0.55758	
39	0.637107	0.259516	0.117314	0.319812	0.512119	0.348523	0.449973	0.357792	arable
54	0.82531	0.588621	0.393353	0.713888	0.706997	0.62585	0.78394	0.667842	
43	0.609853	0.400427	0.225673	0.458572	0.530236	0.454796	0.539279	0.572374	
46	0.806603	0.546066	0.384374	0.71705	0.664334	0.604831	0.752392	0.542179	
31	0.790158	0.591977	0.41167	0.709696	0.678383	0.611934	0.773755	0.619639	
57	0.456022	0.660931	0.580697	0.726048	0.480824	0.601469	0.600324	0.437603	
32	0.562177	0.676842	0.551495	0.718756	0.590976	0.619461	0.614093	0.579831	
45	0.467544	0.423003	0.214324	0.421036	0.429589	0.437654	0.48943	0.587629	
53	0.474722	0.665498	0.62288	0.714923	0.534693	0.578743	0.571937	0.486884	bare land
30	0.634927	0.552087	0.3897	0.621188	0.550485	0.5226	0.703732	0.584108	
24	0.490168	0.644248	0.687665	0.780871	0.516743	0.590439	0.632292	0.414108	
21	0.482945	0.618225	0.562028	0.597282	0.436575	0.61945	0.598841	0.475398	
19	0.396544	0.500852	0.414102	0.392778	0.385138	0.465285	0.398464	0.404823	
9	0.423174	0.55338	0.612084	0.697643	0.429035	0.515147	0.541046	0.351185	
16	0.211442	0.474692	0.590102	0.457264	0.268752	0.410199	0.335801	0.252881	
2	0.373547	0.609212	0.671709	0.715295	0.445892	0.528703	0.542652	0.33978	
12	0.453558	0.405463	0.307176	0.487675	0.37786	0.461018	0.519316	0.374904	beech
14	0.221746	0.44596	0.53973	0.355641	0.270143	0.364864	0.31037	0.171797	
6	0.378031	0.494513	0.573469	0.614519	0.475992	0.474182	0.442344	0.375249	
13	0.347412	0.518811	0.47777	0.390187	0.348045	0.450657	0.413934	0.379168	
22	0.285016	0.548151	0.657674	0.627569	0.376713	0.468019	0.439361	0.291821	
10	0.251168	0.47557	0.630301	0.567722	0.364715	0.410419	0.385139	0.285138	
48	0.637608	0.665996	0.597609	0.83662	0.600185	0.652088	0.737245	0.506114	
49	0.619742	0.622823	0.456074	0.727037	0.563117	0.631964	0.70503	0.668353	grass
5	0.417348	0.599119	0.680211	0.741976	0.505727	0.554437	0.548686	0.375903	
7	0.783401	0.598503	0.449891	0.75481	0.68359	0.636382	0.75897	0.542353	
27	0.711867	0.358672	0.255548	0.483257	0.615233	0.447077	0.561928	0.445182	
28	0.783888	0.52798	0.393221	0.709804	0.655616	0.582254	0.741228	0.494745	
29	0.724523	0.498935	0.300399	0.569256	0.646785	0.527627	0.661745	0.650207	
3	0.668083	0.635537	0.521235	0.799018	0.640535	0.64043	0.71667	0.533046	
26	0.689574	0.562915	0.38515	0.633056	0.622816	0.552837	0.704679	0.625085	olive
59	0.671963	0.566092	0.436732	0.657279	0.664335	0.595557	0.643879	0.523582	
40	0.578896	0.259343	0.136784	0.332147	0.495207	0.351813	0.439867	0.467936	
34	0.470044	0.491017	0.397132	0.529358	0.508869	0.516781	0.457846	0.52119	
35	0.28809	0.486038	0.590508	0.5836	0.427612	0.458406	0.419114	0.281162	
36	0.485922	0.469853	0.379273	0.515377	0.563329	0.481119	0.470894	0.377599	
47	0.793669	0.462266	0.285783	0.601485	0.646086	0.528705	0.684299	0.436621	
8	0.665887	0.568886	0.394432	0.644221	0.577852	0.544451	0.724859	0.606636	
1	0.80877	0.546946	0.388503	0.718146	0.664421	0.60424	0.758571	0.534088	
33	0.510907	0.52117	0.369083	0.545209	0.536263	0.547929	0.491283	0.532157	
41	0.669563	0.483078	0.335585	0.579324	0.561664	0.57761	0.610008	0.841363	
42	0.446169	0.400698	0.232808	0.41044	0.419769	0.47052	0.472019	0.930396	
23	0.764011	0.660052	0.546088	0.824037	0.690094	0.670085	0.793649	0.550561	shrub
44	0.631298	0.575634	0.41455	0.627274	0.564026	0.55702	0.694663	0.584694	
20	0.369716	0.489787	0.394549	0.418266	0.35574	0.53398	0.444183	0.396975	
18	0.267213	0.370611	0.370494	0.338819	0.218164	0.42647	0.340587	0.248719	
37	0.549923	0.597724	0.492308	0.683222	0.597682	0.624028	0.587683	0.568144	
17	0.335732	0.509763	0.554662	0.534041	0.347768	0.527549	0.485879	0.374163	
15	0.496485	0.591986	0.503082	0.633479	0.42889	0.579177	0.597685	0.517594	
50	0.582459	0.627429	0.536024	0.71099	0.635981	0.605942	0.639972	0.48305	
51	0.567069	0.688331	0.603061	0.796908	0.601687	0.647235	0.675754	0.502034	
52	0.64957	0.698218	0.61086	0.841893	0.642748	0.674497	0.735287	0.541804	
4	0.659464	0.596394	0.525522	0.790824	0.582632	0.603365	0.738362	0.513628	
55	0.814406	0.500342	0.333034	0.648159	0.6918	0.570492	0.715362	0.476914	urban
56	0.82369	0.59385	0.42254	0.748838	0.694805	0.634128	0.792009	0.5898	
25	0.640866	0.555725	0.390637	0.610372	0.566858	0.52968	0.69421	0.616016	
58	0.662649	0.602715	0.451524	0.667631	0.663172	0.635498	0.643728	0.692736	
11	0.432502	0.518708	0.438612	0.555104	0.38148	0.535141	0.582006	0.454979	
60	0.722593	0.665242	0.494767	0.791453	0.635008	0.655309	0.80194	0.681049	
38	0.552901	0.496298	0.313547	0.523181	0.496916	0.543267	0.575017	1	wet

Different colors correspond to different environmental cluster, environmental cluster-1 represented by (Pink), environmental cluster-2 represented by (Yellow), environmental cluster-3 represented by (Cyan).
